# Use of a novel pressure distribution system for severely ill neonates: a clinical pilot study carried out by the PREPICare consortium

**DOI:** 10.1186/s12887-023-04252-2

**Published:** 2023-11-23

**Authors:** Anna-Barbara Schlüer, Adrian Yves Müller, Nicolas Philip Fromme, Martin Camenzind, Robert Riener, René Michel Rossi, Barbara Brotschi Aufdenblatten

**Affiliations:** 1 Institute of Nursing, School of Health Professions, Zurich University of Applied Sciences, Winterthur, Switzerland; 2 Division of Neonatology and Pediatric Intensive Care, University Children’s Hospital Zurich, Zurich, Switzerland; 3 Swiss Federal Laboratories for Materials Science and Technology, Laboratory for Biomimetic Membranes and Textiles, Empa, St. Gallen, Switzerland; 4 Sensory-Motor Systems Lab, Dept. of Health Sciences and Technology, ETH Zurich, Zurich, Switzerland; 5 Children’s Research center (CRC) of the University Children’s Hospital Zurich, University of Zurich, Zurich, Switzerland; 6 Medical Faculty, University Hospital Balgrist, University of Zurich, Zurich, Switzerland

**Keywords:** Pediatric Intensive Care Unit, Pressure injuries, Innovation, Prevention

## Abstract

**Background:**

Pressure Injuries are not exclusively an adult phenomenon; various risk factors contribute to a high prevalence rate of 43% in the neonatal and pediatric intensive care population. Effective preventive measures in this population are limited.

**Methods:**

We performed a pilot study to analyze the distribution and localization of support surface interface pressures in neonates in a pediatric intensive care unit (PICU). The hypothesis was that pressure redistribution by a novel air mattress would reduce pressure peaks in critical neonates. The measurements were conducted in a 27-bed level III PICU between November and December 2020. This included measuring pressure distribution and pressure peaks for five neonates positioned on either a state-of-the-art foam mattress or a new prototype air mattress.

**Results:**

We confirmed that the pressure peaks were significantly reduced using the prototype air mattress, compared with the state-of-the-art foam mattress. The reduction of mean pressure values was 9–29%, while the reduction of the highest 10% of pressure values was 23–41%.

**Conclusions:**

The journey to an effective, optimal, and approved product for severely ill neonates to reduce Pressure Injuries is challenging. However, a crucial step was completed by this pilot study with the first pressure measurements in a real-world setting and the successful realization of a decrease in pressure peaks obtained using a prototype air mattress.

## Background

Pressure Injuries (PIs) are not restricted exclusively to adults; it is recognized that children, especially newborns, can also be at high risk [1]. In the general pediatric population, the reported prevalence estimates from the most recent large-scale studies range from 1.4 to 7.1% [2–4]. For more specific subpopulations, namely the pediatric and neonatal intensive care population, prevalence rates as high as 43% and cumulative incidence rates as high as 28.18% have been reported [5, 6]. These high numbers can be explained by the different risk factors contributing to PIs in the neonatal intensive care (NICU) and pediatric intensive care (PICU) populations. Immobility (due to analgosedation/ relaxation) and the resulting constant pressure are risk factors for support surface-related PIs [7, 8]. In neonates minimal handling is an important practice to allow a neonate to be disturbed less. This may lead to prolonged periods of less repositioning which can increase the PI risk. Additionally, due to their general condition, these children often experience rapid and sudden changes in the shape of body sites (e.g., face) due to increased fluid intake, edema, or various medications, which can weaken the skin and increase the pressure further. Furthermore, poor nutritional status due to malnutrition may also negatively affect the integrity of the skin. The use of external devices (e.g., face masks, tubes, and cannulas) as well as decreased tissue tolerance in these patients due to their critical condition means that severely ill children are the most vulnerable to PI’s [9–12]. In the adult population, increased humidity and temperature are important risk factors [9, 13]. However, dehydration and heat loss are of greater concern in the neonatal population and can reduce skin integrity [10, 14], which in turn can favor PI development. Besides provoking pain and stress for the affected individual, PIs also represent a burden for caregivers, as treatment for other illnesses becomes much more complex [7, 8]. The isolated excess costs of PIs in the neonatal population are poorly documented, but, since they can prolong hospital stays, costs are inevitably increased. Goudie et al. reported average excess costs of 85,853 USD per case for patients of 1–4 years of age in the USA [15].

Support surfaces include all types of mattresses, cushions, overlays, and integrated bed systems that provide at least a minimum level of pressure redistribution. In adults, several dynamic and pressure relieving and distributing mattresses are available, based on foam cores, special visco-elastic materials, or air-filled chambers [16]. The pressure distribution and support provided by support surfaces depend on the properties of the foam or the air pressure, as well as the properties of the additional cover layers. However, conventional mattresses are designed for adult patients, to suit a specific body size and weight within a narrow range. Different types of non-gold-standard support surfaces are in use in PICU/NICUs; for example, in the clinic where this study was conducted, various foam mattresses, cushions, rolled cotton towels, and Z-flow fluidized positioners (Mölnlycke Health Care AB) are used. Other approaches can be found in the literature. The air mattress was identified as the most promising choice for this study as finite element simulations by Gefen et al. indicated that air cell-based support surfaces may provide better protection against PIs resulting from cables trapped underneath the body, by dissipating deep-tissue stress states [16]. Moreover, the 2019 PUAP/NPIAP/PPPIA International Pressure Ulcers/Injuries Prevention and Treatment Guideline recommends the use of reactive air mattresses or overlays for the prevention of immobility-related PIs [7, 8]. Air mattresses and more sophisticated devices such as alternating pressure air mattresses are readily available for adults. However, large-scale evidence of their effectiveness is rather weak, as reported by McIness et al. [17].

Mattress systems developed for adult patients do not function well for neonates due to their inappropriate size, structure, and hardness. In addition, they lack sensitivity to the limited weight and sizes of small patients for reactive systems and are unsuitable for the vulnerable skin microclimate of neonates and infants. Given the unique medical environment of PICUs and NICUs, where small beds are often overloaded with medical devices, wiring, tubing, electrodes, and personal items, PI risk seems higher than in the adult population.

## Methods

In this pilot study, we set out to measure the distribution and localization of support surface interface pressures in neonates in a PICU. This included mechanical testing of pressure peaks to compare the state-of-the-art foam mattresses used in the clinics and a new prototype air mattress. This preliminary work was performed in order to develop a prototype mattress to reduce PIs in this patient population. The hypothesis was that pressure distribution is more balanced on an air-filled mattress.

### Setting and patients

The measurements were conducted in the 27-bed level III PICU of a children’s university hospital in Switzerland between November and December 2020. During this period, all regulations with regard to Covid-19 safety measures were followed. During the pressure measurements, every neonate was cared for routinely by the local PICU staff; additionally, experienced PICU-trained nurse supervised the intervention to ensure the comfort of the neonates. The nurse was further in charge of assessing symptoms of discomfort in the neonates, interrupting the measurements if necessary. All measurements were approved by the responsible physician and supervised by medical specialists, while maneuvers with the neonates were carried out exclusively by the medical personnel. Parents of the neonates were invited to accompany their child during the procedure and could interrupt the procedure at any stage. All measurements were conducted in the evening shift between 4 and 10 pm. The measurements were conducted on five neonates who had been admitted to the PICU due to organ failure during their first month of life. During the procedure ventilation and oxygenation levels were constantly assessed in each neonate, and there were no noticeable fluctuations.

### Ethical approval

The pilot study was assessed by the ethics committee of the hospital as well as all involved parties within the PREPICare consortium (BASEC 2020 − 01183; ETH: EK 2020-N-153). Informed consent was provided by the parents.

### Interventions and materials

PIs are defined by the European Pressure Ulcer Advisory Panel (EPUAP), the National Pressure Injury Advisory Panel (NPIAP, formerly National Pressure Ulcer Advisory Panel), and the Pan Pacific Pressure Injury Alliance (PPPIA) as.

*…localized damage to the skin and underlying soft tissue usually over a bony prominence or related to medical or other devices. The injury can be present on intact skin or as an open ulcer and may be painful. The injury occurs as a result of intense and/or prolonged pressure or pressure in combination with shear. The tolerance of soft tissue for pressure and shear may be affected by microclimate, nutrition, perfusion, comorbidities, and condition of the soft tissue.* [7, 8]

This general definition defines all PI types and encompasses various causal factors.

We measured pressure distribution for a standard foam mattress (Babytherm 8004, Dräger, Lübeck, Germany) as well as for the prototype of a laser-welded air-filled mattress developed by Empa, Swiss Laboratories for Materials Science and Technology, St. Gallen, Switzerland.

**Fig. 1:**
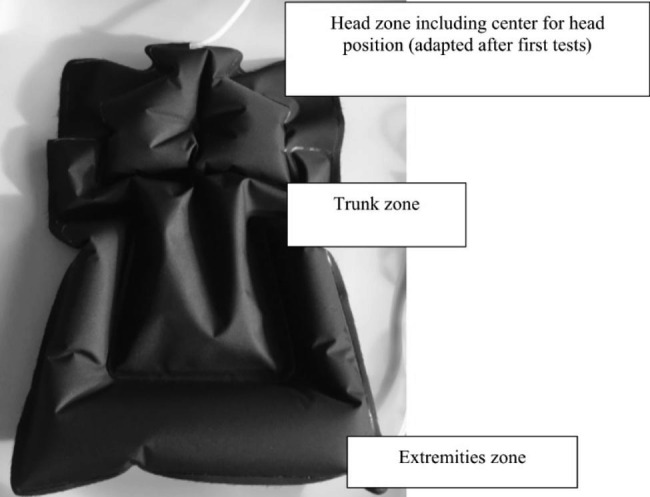
The inflated air mattress with labeling of the three different zones connected to the air tubes

Air mattress prototype development: laser welding (as described previously by Fromme et al. [18]) was used to form air-filled structures based on thermoplastic polymers. Polyvinylchloride (PVC)/ polyurethane (PUR) angle connectors (Carmo A/S, Espergærde, Denmark) were welded into each chamber to allow connection to tubes for inflation of the created 3D structures. An iterative approach to prototyping and testing in the lab was used to optimize the structure for testing in the clinical setting. The structure was formed of three unconnected air chambers, also referred to as zones. Different types of membranes and laminates were explored, with the final version being made from a Sympatex polyester laminate (Sympatex 150 STX Orinoco, Sympatex Technologies GmbH, Unterföhring, Germany) (Fig. 1). An electronic unit consisting of a microprocessor, pump, several valves, and multiple pressure sensors allowed for exact pressure regulation in the different zones, the in- and deflation process was performed silently. The material is approved for the use in PICU settings, and can be disinfected according to standard hospital procedures.

### Measurement set-up

The air mattress was placed on top of a standard foam mattress for the measurements. Numeric analysis of the pressure peaks was carried out, and representative MATLAB color maps were produced for visual comparison of the deflated and inflated states.

A flexible pressure-sensing mat (PSM; Xsensor LX100:40.40.02, XSENSOR Technology Corporation, Calgary, Canada) with a matrix of 40 × 40 sensing units and spanning an area of 508 mm x 508 mm was used for the pressure measurements. The measurement range was 5–200 mmHg, with an accuracy of 10% in this range; values below 5 mmHg were excluded automatically by the electronics and software of the PSM.

Pressure distributions for the neonates on the mattress system were measured on-site. Before the start of the experiment, we set the baseline by lying the neonates on a standard foam mattress (Babytherm 8004) with all other positioners (e.g., towels) removed. A thin medical sheet (curea medical GmbH, Steinfurt, Germany) was allowed for the capture of bodily fluids and protection of both the baby (in diapers) and the sensor mat (PSM). The measured interface pressures were constantly monitored by computer. The neonates were in a supine position for all measurements. The measurements were split into four sections, and four minutes of data were collected for each section.

The measurements taken for the first neonate also served as verification of the setup, as initial testing of the air mattress could only be carried out using a baby doll. As a result of the first measurement with a neonate, a small adaptation was made to the air mattress: a small circular seam at the center of the head zone was welded into the mattress to decrease central bloating in this chamber and stabilize the baby’s head (Fig. 1). The future proposition is to use the mattress in an adaptable but steady-state performance with only slight inflation changes.

Sequence of measurements to assess pressure distributions (four minutes of data collected per stage) :

- Baseline (sequence 1): the nursing staff positioned the neonate on the standard foam mattress equipped with the PSM and the medical sheet. The PSM was checked for potential folds or overstretched areas.
- Air mattress, deflated (sequence 2): the neonate was lifted, together with the medical sheet and PSM, and the air mattress was positioned in between the PSM and the standard foam mattress in a deflated state.
- Inflation of air mattress (sequence 3): the air mattress was inflated gradually, zone by zone, in multiple cycles, until the neonate was markedly raised. This inflation took up to 10 min. With the neonate in a supine position, body parts in the three different zones remained horizontally aligned.
- Air mattress, steady state (sequence 4): after the inflation state was reached, another four-minute steady-state measurement was taken.

### Data and statistical analysis

The pressure data captured by the PSM was registered with a sampling frequency of 5 Hz, and MATLAB software was used for data processing and analysis. The 40 × 40 value matrices corresponding to the measurement frames were saved in 3D structures for analysis (40 × 40 x time). Illustration of the pressure data by colormaps allowed easy recognition of zones with elevated pressure. The colormaps corresponded to the data matrices, whereby the pressure value of every sensel was represented by a colored square according to pressure level. Histograms were used to show the differences in pressure value frequencies for the entire body and the occiput in particular. Boxplots were used to compare the baseline and steady states for all neonates; the first box plot included all values ≥ 5 mmHg, while a second box plot included only the highest 10% of interface pressure values (representing the potentially problematic peak pressure values). This percentage was arbitrarily defined since there is no reference for “high” interface pressures for neonates, as mentioned previously [16].

Outliers in the box plot are defined as values 1.5 times higher or lower than the maximum or minimum values in the interquartile range, respectively. Boxes whose notches (median line) did not overlap had dissimilar medians at a 5% significance level. For a non-visual statistical analysis, the mean values of pressures per neonate and sequence were calculated and compared. The Wilcoxon signed rank-sum-test was used to test the significance of the effect at a 5% significance level. This analysis was performed for both data sets, i.e., the full set and the set including the highest 10% of values only. Percentage differences between the baseline and steady-state means for each neonate were calculated to show the size of the effect.

## Results

### Study population

Pressure measurements were performed for five neonates in November and December 2020. The demographic data of the study population are provided in Table 1.

**Table 1:** Demographic data of the study population

	Neonate 1	Neonate 2	Neonate 3	Neonate 4	Neonate 5
Postnatal age (days)	23	5	4	3	4
Gender	male	female	female	male	male
Gestation age at birth (weeks)	37 1/7 GW	31 2/7 GW	36 4/7 GW	35 2/7 GW	38 0/7 GW
Ethnic group	white	white	white	white	white
Weight (kg)	3.43	1.725	2.74	2.13	3.22
Length (cm)	49.5	46	48	44	48
Head circumference (cm)	33	31	30	32	36
Analgosedation (yes/no)	no	yes	yes	yes	yes
Ventilation (yes/no including type)	yes CPAP	yes CPAP	yes invasive ventilation	yes invasive ventilation	yes invasive ventilation

CPAP: continuous positive pressure ventilation with a nasal mask

GW: gestational weeks

Invasive ventilation: invasive ventilation with a tracheal tube

### Pressure measurements

**Fig. 2:**
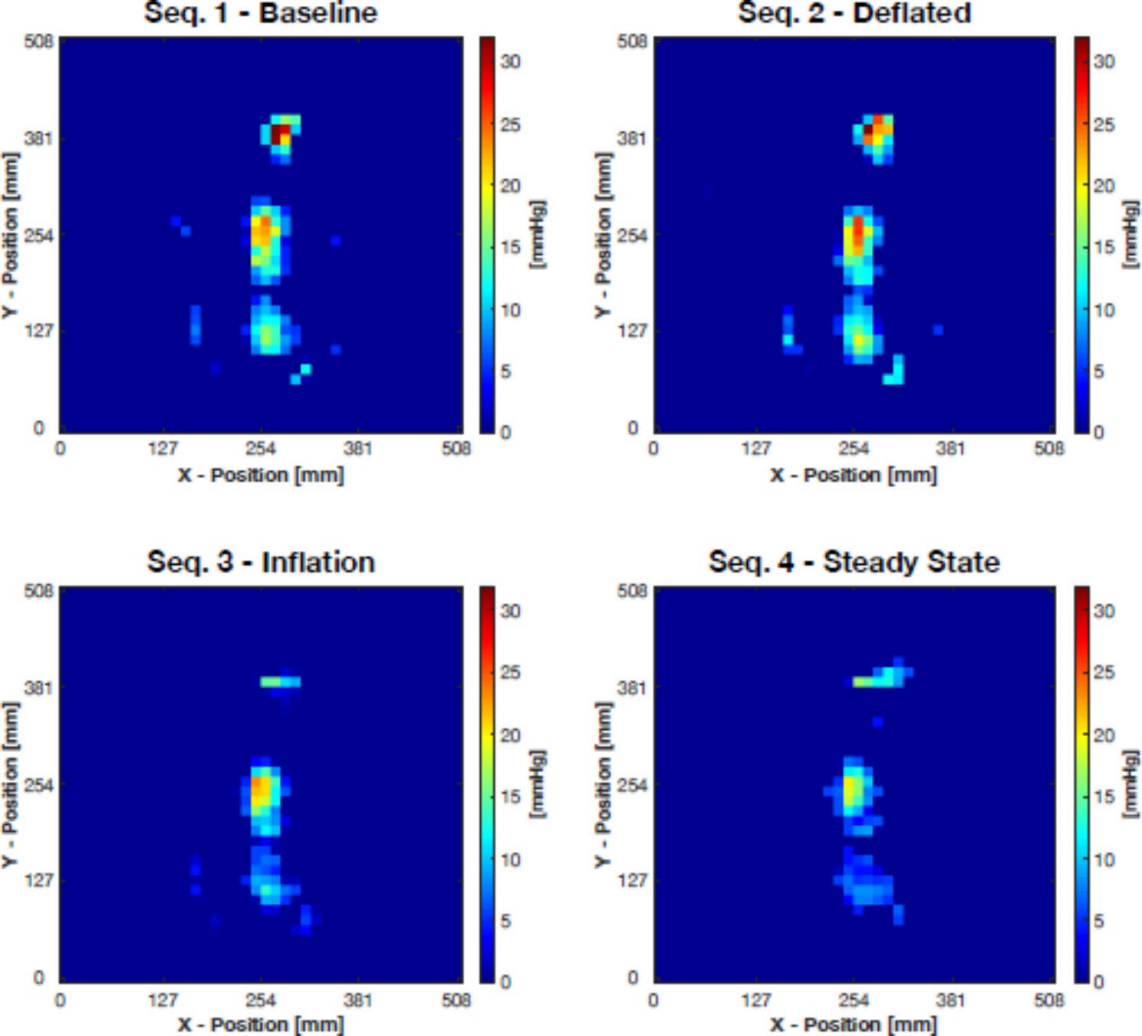
Pressure maps for neonate number 5. The average of all frames is displayed for each sequence. Measurement times were between four and six minutes, due to the neonates’ periods of activity. Pressure zones for the head, shoulders, and sacrum can be seen (from top to bottom in the images), while the extremities are also partly visible in the baseline sequence image as a result of slightly higher pressures

Neonate 2 showed some activity during the inflation sequence and had to be repositioned multiple times. However, a period of calmness followed, and the steady-state sequence was not affected by movement. The other neonates (neonates 1, 3, 4, and 5) showed negligible levels of activity, and their body positions remained stable. In the pressure maps, pressure areas for the head, shoulders, and sacrum can be distinguished for all neonates. Figure 2 shows neonate 5 as a representative example; the extremities can also be faintly distinguished in the baseline sequence pressure map. For all five neonates, the highest pressures were measured at the occiput, followed by the shoulders. Generally, the pressures were higher during the deflated sequence than during the baseline sequence.

**Fig. 3:**
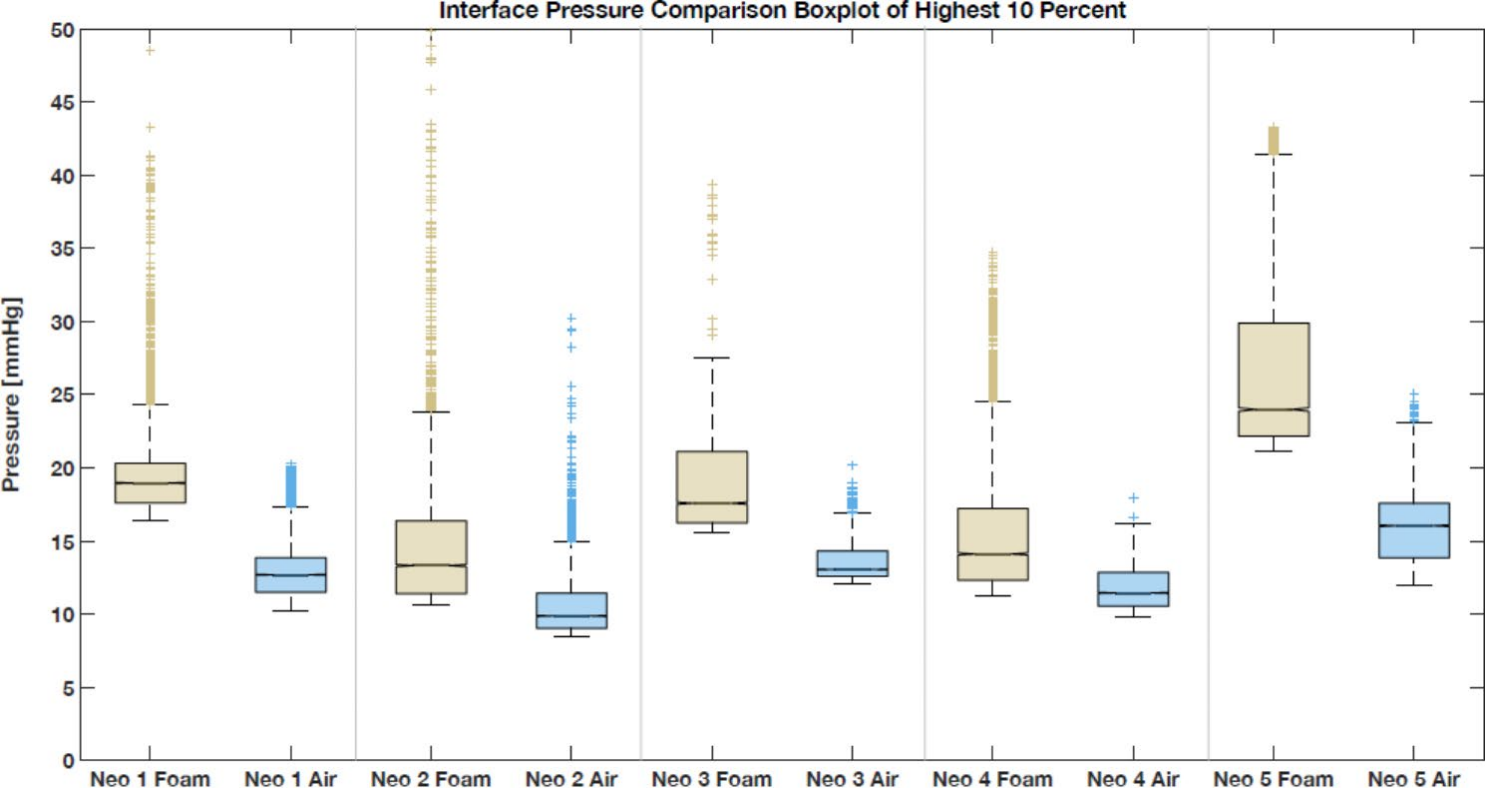
Box plot of the top 10% of pressure values for the baseline sequence (standard foam mattress) and steady-state sequence (air mattress) for all neonates. Notches in the box around the median indicate dissimilar medians for the sample at the 5% significance level, if not overlapping. Due to the large amount of data, the notches are too small to be visible but do not overlap

For all neonates, the mean steady-state sequence interface pressures were significantly reduced, compared with baseline values, with differences ranging from 9.4 to 29.2% (p = 0.03; Table 2). In addition, the differences were greater for the highest 10% of steady-state sequence interface pressure values (number of values 𝒗 = 9451) in all neonates; significant reductions ranging from 22.7 to 40.8% of baseline pressure values were observed (p = 0.03; Fig. 3).

**Table 2:** Pressure means for baseline and steady-state sequences

p = 0.03	neonate 1	neonate 2	neonate 3	neonate 4	neonate 5
Mean pressure values for baseline and steady-state					
Baseline (mmHg)	8.3	8.8	9.6	8.8	11.8
Steady-state (mmHg)	7.5	7.2	8.0	6.8	8.3
Difference in %	9.4	18.0	16.6	22.7	29.2

## Discussion

In this pilot study, we measured the support surface interface pressure distribution for critically ill neonates on a standard foam mattress and compared the resulting pressure peaks with those obtained using a newly developed prototype air mattress. We confirmed that the pressure peaks were significantly reduced with the prototype air mattress, compared with the standard foam mattress. The reduction of mean pressure values was 9–29%, while the highest 10% of pressure values were reduced even further, by 23–41%. The areas of pressure peaks identified in our study population (head, shoulders, and sacrum) correspond with the reported PI locations for this population [1–5, 19, 20]. Thus, the data of this preliminary work may support the assumption that using an air-filled mattresses may prevent PIs in critically ill infants.

An effective air mattress prototype was successfully produced by Empa (Switzerland) using a laser-welding technique. This technique allows the rapid and precise manufacture and adaptation of designs in 2D and is thus optimal for prototyping. However, predicting the shape of the inflated 3D structure is challenging, while the production of cubic structures (geometrically the most useful for mattress design) to meet the specific needs of the population of this study was challenging. To the best of our knowledge, this was the first time that support surface interface pressures have been measured in order to compare and quantify pressure means and peaks in a clinical setting. Trials with similar devices in the pediatric population are very scarce. In their observational and descriptive prospective longitudinal (2009–2011) study of a pediatric-specific, continuous and reactive low-pressure mattress, Garcia-Molina et al. reported a decrease in PI incidence of 16.7% [21]; however, they did not differentiate the pressure interface means and peaks. In another NICU-based study carried out by Aziz et al. [22], the aim was to detect patient movements in order to monitor signals that may indicate patient false alarms. Our pressure maps appeared similar to those published by Aziz et al. [22]; for example, the head, shoulders, and sacrum (referred to as “legs” by Aziz) are distinguishable in both studies. Aziz et al. used a PSM made by the same manufacturer (albeit with a higher resolution); however, their study focused on the detection of the center of pressure distribution and did not discuss pressure peaks or magnitudes. Future work would certainly benefit from measurements carried out using a higher resolution mattress, which would allow pressure peaks to be identified more accurately, both spatially and quantitatively. At the time we conducted this pilot study, the PSM used by Aziz et al. [22] was not available.

Apart from the pressure measurements, different information for design optimization was gathered. With the present design, seams between the different zones were inevitable and posed problems, especially at the neck. Therefore, apart from alternative ways of fabricating cubic structures, multi-layered approaches could also be investigated. In future designs, the interdependency of multiple variables will have to be investigated systematically as they cannot be transferred from designs developed for the adult population. One particular challenge is to ensure a certain transfer of water vapor through the mattress, as moisture is a key factor in PI development [9]. Active control of temperature and moisture levels would thus be desirable, which could be regulated via the flow of conditioned air in the chambers in the future. However, for neonates, the role of moisture is ambiguous, and the functionality of a potentially moisture-regulating structure would need to be explored in relation to PI risk. Transepidermal water loss (TEWL) changes dramatically within the first weeks of life and might have an even higher impact on preterm neonates. To explore this further, investigation regarding the impact of TEWL in term and preterm neonates in real-life situations is required [9, 10].

The measured pressure peaks in the head zone during the baseline sequences were in agreement with studies reporting the occiput as a prominent PI risk zone [14, 16, 14, 23]. However, the shoulders, which are rarely reported as a PI location in the literature, represented the areas with the second biggest pressure peaks in our measurements. Clinical experts highlight the softness of the shoulder structures in neonates as a possible explanation. In addition, the pelvis and sacrum are locations that have been shown to be generally affected by PI in the pediatric population [7, 24]. It is possible that diapers might act as a buffer for this region; however, more detailed analysis of this would require removing the diaper and measuring the pressure on the skin directly, which would still not be fully accurate unless pressures were measured within the diaper. As for the absolute range of pressures detected, this differed among the measured neonates, while analysis of five individuals is insufficient to make a generalizable statement. Nevertheless, the range of means of the highest 10% of baseline sequence values (see Fig. 3) provides a reasonable estimate, showing pressure peaks in the range of 15–27 mmHg. Analysis of absolute pressure values in relation to the weight of the subjects may also be informative. The air mattress allows adaptation to the different weights of neonates and infants, in contrast to a standard foam mattress. However, since increased weight leads to increased immersion, area of contact, and thus pressure redistribution, the relationship between weight and pressure may be complex. Factors like muscle tonus, activity, postnatal age, and skin edema may also have a sizable influence. A very general limitation is that interface pressures fall short of capturing actual deep tissue stress states [20, 25]. Nevertheless, interface pressures are currently the best-known indicator for neonates. The high variability of physiological and anatomical parameters in neonates treated in PICUs (using the weights from Table 1 as an example) makes a one-size-fits-all solution illusory. Conversely, providing a support surface with a high degree of individual specification represents a complex challenge, as the target group is already very specific and thus small.

Our study has some limitations. As mentioned, the standard foam mattress placed underneath the air mattress had the potential to affect interface pressure measurements when the foam mattress was relaxing or otherwise deforming. The foam mattress was required for safety reasons, in case the air mattress deflated accidentally. However, once a steady state was reached and the air mattress contained enough air to fully lift the neonate’s body, the foam mattress had no further influence on the pressures measured by the sensor mat at the interface of the air mattress and the neonate’s body. Moreover, the applied sensor mat was not sensitive enough to detect pressure distribution changes of this magnitude. The patient sheet could also be viewed as a possible confounding factor, as it might also have had a slight pressure redistributive impact; furthermore, this may have been more significant for the uneven air mattress surface than the more planar foam mattress.

Possible future trials with healthy neonates should also be considered, for improved accessibility and statistics. However, this may lead to issues of comparability due to excessive movement, while exploring causality would not be possible as healthy neonates would be less vulnerable to PI development than this very small study population.

## Conclusion

To the best of our knowledge, these are the first measurements of support surface-related interface pressures in severely ill neonates in a PICU to study risk zones for PI development. A novel air mattress prototype was developed and studied as a support surface for clinical settings. It was successfully shown that the air mattress redistributed pressure forces and significantly decreased pressure peaks, in comparison with a standard foam mattress. The comparison of pressure values measured on the state-of-the-art foam mattress and the air mattress proved that the pressure values shifted to lower magnitudes for the air mattress prototype. A crucial step for the development of air-filled mattresses for the PICU setting was completed with this pilot study, showing for the first time the relevance of pressure measurements in a real-world setting and providing practical design information. Overall the very low number of infants analyzed in the study is a major limitation and does not allow any generalizable findings. None the less, the study shows pressure distribution in a very unique and vulnerable setting of neonates with paucity of evidence.

## Data Availability

The datasets used and/or analysed during the current study available from the corresponding author on reasonable request.

## Abbreviations

PIs: Pressure Injuries
NICU: Neonatal Intensive Care Unit
PICU: Pediatric Intensive Care Unit
EPUAP: European Pressure Ulcer Advisory Panel
NPIAP, formerly National Pressure Ulcer Advisory Panel: National Pressure Injury Advisory Panel
PPPIA: Pan Pacific Pressure Injury Alliance
PVC: Polyvinylchloride
PUR: Polyurethane
PSM: Pressure-sensing Mat
TEWL: Transepidermal Water Loss

## References

[1] Delmore B (2019). Pressure injuries in the Pediatric Population: a national pressure Ulcer Advisory Panel White Paper. Adv Skin Wound Care.

[2] Pellegrino DMS (2017). Prevalence and incidence of pressure injuries in pediatric hospitals in the city of Sao Paulo, SP, Brazil. J Tissue Viability.

[3] Razmus I, Bergquist-Beringer S (2017). Pressure Injury Prevalence and the rate of Hospital-Acquired pressure Injury among Pediatric Patients in Acute Care. J Wound Ostomy Continence Nurs.

[4] Sanchez-Lorente MM (2018). Prevalence of pressure ulcers in the paediatric population and in primary health care: an epidemiological study conducted in Spain. J Tissue Viability.

[5] García-Molina P, Balaguer-López E, García-Fernández FP, Ferrera-Fernández MLÁ, Blasco JM, Verdú J (2018). Pressure ulcers’ incidence, preventive measures, and risk factors in neonatal intensive care and intermediate care units. Int Wound J.

[6] Schluer AB, Halfens RJ, Schols JM (2012). Pediatric pressure ulcer prevalence: a multicenter, crosssectional, point prevalence study in Switzerland. Ostomy Wound Manage.

[7] Haesler E, European Pressure Ulcer Advisory Panel (EPUAP), National Pressure Ulcer Advisory Panel (NPUAP) (2014). Pan Pacific pressure Injury Alliance (PPPIA). Prevention and treatment of pressure ulcers: quick reference guide.

[8] National Pressure Ulcer Advisory Panel (NPUAP). Pressure Injury stages. 2016. https://tinyurl.com/tu3kjwh (accessed February 5, 2020).

[9] Schwartz D (2018). Effects of humidity on skin friction against medical textiles as related to prevention of pressure injuries. Int Wound J.

[10] Oranges T, Dini V, Romanelli M (2015). Skin physiology of the neonate and infant: clinical implications. Adv Wound Care (New Rochelle).

[11] Curley M, Quigley S, Lin M (2003). Pressure ulcers in pediatric intensive care: incidence and associated factors. Pediatr Crit Care Med.

[12] Noonan C, Quigley S, Curley M (2006). Skin integrity in hospitalized infants and children: a prevalence survey. J Pediatr Nurs.

[13] Zeevi T (2018). Effects of ambient conditions on the risk of pressure injuries in bedridden patients-multi-physics modelling of microclimate. Int Wound J.

[14] Baharestani MM, Ratliff CR (2007). Pressure ulcers in neonates and children: an NPUAP white paper. Adv Skin Wound Care.

[15] Goudie A (2015). Costs of venous thromboembolism, Catheter-Associated urinary tract infection, and pressure Ulcer. Pediatrics.

[16] Levy A, Kopplin K, Gefen A (2015). Adjustability and adaptability are critical characteristics of Pediatric Support Surfaces. Adv Wound Care (New Rochelle).

[17] McInnes E (2018). Support surfaces for treating pressure ulcers. Cochrane Database Syst Rev.

[18] Fromme NP (2020). Design of a lightweight passive orthosis for tremor suppression. J Neuroeng Rehabil.

[19] Visscher M, Taylor T (2014). Pressure ulcers in the hospitalized neonate: rates and risk factors. Sci Rep.

[20] Levy A, Kopplin K, Gefen A (2017). Device-related pressure ulcers from a biomechanical perspective. J Tissue Viability.

[21] Garcia-Molina P (2012). A prospective, longitudinal study to assess use of continuous and reactive low-pressure mattresses to reduce pressure ulcer incidence in a pediatric intensive care unit. Ostomy Wound Manage.

[22] Aziz S et al. Detection of Neonatal Patient Motion Using a Pressure-Sensitive Mat, in 2020 IEEE International Symposium on Medical Measurements and Applications (MeMeA). 2020: Bari, Italy. p.1–6.

[23] Manning MJ, Gauvreau K, Curley MA. Factors Associated With Occipital Pressure Ulcers in Hospitalized Infants and Children. Am J Crit Care. 2015;24(4):342-8. doi: 10.4037/ajcc2015349. PMID: 26134335. 10.4037/ajcc2015349 26134335

[24] Kottner J, Wilborn D, Dassen T (2010). Frequency of pressure ulcers in the paediatric population: a literature review and new empirical data. Int J Nurs Stud.

[25] Gefen A, Levine J (2007). The false premise in measuring body-support interface pressures for preventing serious pressure ulcers. J Med Eng Technol.

